# Unidirectional rating scales overestimate the illusory causation phenomenon

**DOI:** 10.1177/17470218231175003

**Published:** 2023-05-23

**Authors:** David W Ng, Jessica C Lee, Peter F Lovibond

**Affiliations:** UNSW Sydney, Sydney, NSW, Australia

**Keywords:** Illusory causation, causal learning, rating scale, contingency learning, associative learning, causal reasoning

## Abstract

Illusory causation is a phenomenon in which people mistakenly perceive a causal relationship between a cue and outcome even though the contingency between them is actually zero. Illusory causation studies typically use a unidirectional causal rating scale, where one endpoint refers to no relationship and the other to a strongly positive causal relationship. This procedure may bias mean causal ratings in a positive direction, either by censoring negative ratings or by discouraging participants from giving the normative rating of zero which is at the bottom extreme of the scale. To test this possibility, we ran two experiments that directly compared the magnitude of causal illusions when assessed with a unidirectional (zero—positive) versus a bidirectional (negative—zero—positive) rating scale. Experiment 1 used high cue and outcome densities (both 75%), whereas Experiment 2 used neutral cue and outcome densities (both 50%). Across both experiments, we observed a larger illusory causation effect in the unidirectional group compared with the bidirectional group, despite both groups experiencing the same training trials. The causal illusions in Experiment 2 were observed despite participants accurately learning the conditional probabilities of the outcome occurring in both the presence and absence of the cue, suggesting that the illusion is driven by the inability to accurately integrate conditional probabilities to infer causal relationships. Our results indicate that although illusory causation is a genuine phenomenon that is observable with either a undirectional or a bidirectional rating scale, its magnitude may be overestimated when unidirectional rating scales are used.

## Introduction

The ability to learn causal relationships between events is crucial to how we interact with the world. An essential component of assessing causal relationships is to determine the contingency between events: how regularly and reliably one event follows another. For example, in determining whether a medicine causes recovery from a headache, one would compare the likelihood of recovery when they took the medicine to the likelihood when they did not take the medicine. If recovery occurred more often when medicine was taken compared to when it was not, one would infer a *positive* contingency, and that taking the medicine *causes* recovery. Conversely, recovery occurring *less* often when taking the medicine implies a *negative* contingency, and that taking medicine *prevents* recovery. An interesting case exists when there is an equal likelihood of recovery after taking medicine compared to not taking medicine. There is a *null contingency* between these events, implying that there is no causal relationship between taking the medicine and recovery.

Generally, people are sensitive to distinguishing between different positive and negative contingencies (Shanks & Dickinson, 1987; Wasserman, 1990). However, people seem to have difficulty when learning about null contingencies and can develop the false belief that random events are causally related (Alloy & Abramson, 1979). This phenomenon, named illusory causation, has been replicated in a variety of contingency learning tasks (see Matute et al., 2015 for review). A common feature of experiments that generate the illusory causation effect is their use of high cue and outcome densities in their designs. That is, there is a higher ratio of trials where the cue is present compared with trials where the cue is absent (Blanco et al., 2013; Hannah & Beneteau, 2009; Perales et al., 2005) as well as a higher occurrence of trials where the outcome is present (Allan & Jenkins, 1983; Jenkins & Ward, 1965; Vallee-Tourangeau et al., 2005), both of which have been shown to increase the magnitude of causal illusions. Illusory causation is particularly interesting as it provides an in-lab analogue of how people may form fallacious beliefs in real-world contexts with high cue and outcome density, such as the efficacy of pseudomedicines (Torres et al., 2020), stereotype formation (Hamilton & Gifford, 1976), and superstitious beliefs (Blanco et al., 2015). For example, homoeopathic pills can be taken frequently as they are readily available, allowing for a high cue density environment. Furthermore, medical conditions such as headaches typically fluctuate in magnitude and are short-lasting, thus providing a high outcome density. As such, the efficacy of pseudomedicines such as homoeopathic treatments can be conflated with these natural recoveries, leading to a high density of misattributed recovery outcomes.

Experiments investigating illusory causation are commonly conceptualised in terms of a 2 × 2 contingency table, which classifies trials by the presence and absence of a binary cue (potential cause) and a binary outcome (see Table 1). Framing trials in such a way allows experimenters to calculate and manipulate Δ*P* (Equation 1), which is an objective measure of contingency between a cue and outcome. Δ*P* is derived from the difference in conditional probabilities between the outcome occurring when the cue is present compared with when the cue is absent (Jenkins & Ward, 1965; Ward & Jenkins, 1965). A positive Δ*P* indicates a positive contingency, whereas a negative Δ*P* indicates a negative contingency and a Δ*P* equal to zero indicates a null contingency. Participants’ causal judgements often track Δ*P* accurately in contingency learning studies (Shanks & Dickinson, 1987; Wasserman, 1990). In the illusory causation task, however, participants report positive causal judgements even though Δ*P* is zero (e.g., Blanco et al., 2013; Chow et al., 2019), which is a non-normative judgement.

**Table 1.** Typical 2 × 2 contingency table used to design causal learning experiments.

	Outcome present	Outcome absent
Cue present	*A*	*B*
Cue absent	*C*	*D*

(1) $$\Delta P\;=\frac{a}{a+b}-\frac{c}{c+d}$$

A common feature of illusory causation experiments is the use of unidirectional scales to measure participants’ causal judgements. A typical causal rating question with a unidirectional scale involves asking participants to indicate how effective the cue is at producing an outcome by choosing numerical values on a labelled slider scale ranging from 0 (not effective at all) to 100 (totally effective; e.g., Barberia et al., 2019; Blanco et al., 2011; Matute et al., 2011). A bidirectional scale, on the other hand, extends the unidirectional scale to include both causal and preventive (e.g., 100; totally preventive) judgements. Blanco and Matute (2020) argue against the use of bidirectional scales in favour of unidirectional scales in illusory causation experiments. Many illusory causation experiments present a medical scenario instructing participants to assume the role of a patient or doctor in a clinical trial and to observe and determine the effectiveness of an experimental drug (Barberia et al., 2019; Chow et al., 2019; Matute et al., 2011; Yarritu et al., 2014). Blanco and Matute (2020) posit that participants may be confused by the idea that medicine could worsen symptoms. However, it is not difficult to imagine a situation where an experimental drug may not only be ineffective but also potentially prevent a patient’s recovery. In fact, Lee and Lovibond (2021) have demonstrated that participants can learn and report preventive relationships in a food allergist paradigm despite the counterintuitive concept of foods preventing allergic reactions.

There are several issues that may arise when using unidirectional rating scales to measure causal judgements and illusory causation. First, unidirectional scales do not allow participants to report preventive relationships. By effectively censoring one section of the distribution of participants’ possible causal beliefs, unidirectional scales are potentially biasing the mean rating in a positive direction. It has also been demonstrated that participants are often reluctant to make responses at the extremes of rating scales (see Baumgartner & Steenkamp, 2001 for review). This is especially problematic for studies of illusory causation in which the normative rating is zero (i.e., no relationship), which lies at the bottom extreme of a unidirectional scale. In a study directly comparing rating scales, Neunaber and Wasserman (1986) found that presenting participants with a bidirectional scale in an instrumental contingency learning task led to more accurate and sensitive estimates of contingencies (including null contingencies) compared to unidirectional scales, and participants were less biased by outcome density effects. The bidirectional scale points ranged from preventive to causal, whereas the unidirectional scale ranged from “no control” to “complete control” over the outcome. Interestingly, participants under the unidirectional condition made more accurate estimates of contingencies when presented with additional instructions that the relationship between their actions and the target outcome could be either causal or preventive. This implies that participants may not spontaneously consider the full range of potential relationships when making causal judgements on a unidirectional scale. Applying these findings to illusory causation paradigms, unidirectional scales may therefore bias participants who would have correctly rated the contingency as null towards reporting positive responses. Furthermore, participants who believe there is a small positive relationship may be encouraged to report even more positive responses, which could overestimate the actual size of the illusory causation effect.

Despite previous studies having already investigated potential problems regarding the use of undirectional scales in causal learning tasks, some issues still remain unresolved. Neunaber and Wasserman (1986) directly compared bidirectional and unidirectional scales, but they presented participants with multiple contingency problems in a within-subjects design. Participants tend to perform more accurately in contingency learning tasks when presented with multiple contingency problems including null contingencies (Allan & Jenkins, 1980; Neunaber & Wasserman, 1986; Shanks & Dickinson, 1987). This improved performance is likely due to participants benefitting from the opportunity to compare different contingencies and calibrate their causal judgements in these tasks. Studies of illusory causation, by contrast, tend to use between-subjects designs (Chow et al., 2019; Matute et al., 2011; Vadillo et al., 2013), as they are primarily interested in investigating conditions that produce and eliminate causal illusions. These studies often use a standard group trained with a single null contingency as a control to compare with a group that receives a manipulation designed to reduce causal illusions.

Interestingly, Blanco et al. (2013) also found that participants judged null contingency problems more accurately when tested on a bidirectional rating scale. They surprisingly observed a positive bias for a non-contingent cue and outcome pair in a low (20%) cue and outcome density contingency learning task when recording causal judgements with a unidirectional rating scale. This effect did not replicate in a following experiment, where participants tested on a bidirectional scale gave more normative responses (i.e., causal ratings close to 0) for a low cue and outcome density null contingency problem. Similarly, Blanco and Matute (2020) compared a procedure modelling pseudotherapy for a spontaneously recovering disease with a standard illusory causation paradigm typically used as a control group. They could only observe causal illusions in their control group with a unidirectional scale, whereas the effect disappeared in a follow-up experiment using a bidirectional scale. The results of these two studies imply that participants may have genuinely believed a non-contingent cue and outcome were unrelated but were biased towards making positive causal judgements on a unidirectional rating scale. However, Blanco et al. (2013) and Blanco and Matute (2020) did not aim to investigate the influence of rating scales and additional methodological changes between experiments (e.g., task cover story) complicate cross-experiment comparisons to make inferences about potential biases of unidirectional scales.

Given that illusory causation paradigms are used to explain how people may misinterpret causal relationships, it is crucial that experiments use an unbiased and sensitive measurement to investigate this phenomenon. As the overall aim of such studies is to eventually identify conditions that can eliminate causal illusions in real-world contexts, we need to ensure first that we are accurately measuring the strength of causal illusions in lab paradigms. Overestimating participants’ causal judgements in illusory causation studies can lead to misinterpretations from combining participants who are unsure or believe there is a null contingency with other participants who genuinely hold an erroneous belief that a causal relationship exists.

## Experiment 1

Experiment 1 aimed to determine whether the effect of rating scales extends to illusory causation paradigms, in which a contingency bias is induced by high cue and outcome density. We tested this by directly comparing independent groups of participants given either a bidirectional or unidirectional response scale in an illusory causation paradigm with a null contingency. Although previous studies have observed differences in causal judgements for contingency problems when measuring with bidirectional and unidirectional rating scales, this experiment will directly compare how presenting these rating scales can affect causal judgements in the same causal scenario with a single null contingency problem. Presenting a single contingency problem prevents participants from comparing different contingencies within the same task to calibrate their judgements and allows us to isolate the potential impact of the rating scales. We hypothesised that participants would report stronger causal relationships between the cue and outcome when presented with a unidirectional scale compared to a bidirectional scale at test despite experiencing the same null contingencies during training.

### Method

#### Participants

This study was conducted online with participants recruited from the online platform Prolific. Participants were required to be fluent in English and have a minimum Prolific approval rate of 90%. A total of 119 participants were recruited (48 female, 69 male, 2 non-binary, M age = 27.87, *SD* age = 9.45). This sample size was chosen to be comparable to previous studies of illusory causation (Blanco & Matute, 2020; Blanco et al., 2020; Chow et al., 2019). We intended participants to take 15 min to complete the experiment and they were paid £1.88 for participating (a rate of £7.52/hr), although the median task completion time at the end of testing was 9 min 38 s. Participants were recruited until at least 50 participants were randomly allocated into the unidirectional or the bidirectional group.

#### Materials

The experiment was coded in Javascript using the jsPsych library (De Leeuw, 2015) and was hosted on a JATOS server for online distribution and data storage (Lange et al., 2015). Participants were required to complete the experiment on their personal desktop computers. The stimuli used were 200 × 200 px images representing a light in an on (blue circle) position and an off position (grey circle), as well as a 250 × 250 px electric shock symbol. The stimuli, experimental code and Supplementary Materials can be found at https://osf.io/3ukbp/.

#### Procedure

This study was approved by the University of New South Wales Human Research Ethics Committee (HREAP #3316). Before starting the task, participants were presented an online information statement with broad-level details of the overall study. Participants declared consent to the experiment by selecting a checkbox to acknowledge they had read and understood the information statement and would like to participate in the study. In the experimental task, participants were presented with a hypothetical scenario in which a fictional Mr. X is investigating a strange machine that delivers electric shocks. They were told that the machine also has a light that periodically turns on and off, and were shown what the light looks like when on (blue) and off (grey). Participants were instructed to observe instances when the light was either on or off and a shock either occurred or not, to help Mr. X determine whether there is any relationship between the light and shock (see Supplementary Materials). This cover story was chosen over a medical scenario typically used in illusory causation studies (e.g. Matute et al., 2011), where the cue is the administration of medicine and the outcome is recovery from a disease. The task instructions used in these medical scenarios often focus on whether the medicine cures participants from a target disease, which may potentially bias participants towards expecting a positive relationship. The light-shock scenario was intended to be more neutral by not hinting at the nature of the relationship (if any) between the light and shock and so could be less likely to bias participants into believing a positive causal relationship existed. The instructions were followed by an instruction check, in which participants were asked to identify the correct task instructions, as well as identify the light in the off position. If participants answered any questions incorrectly, they were timed out for 5s before being allowed to attempt the questions again. Participants were only able to proceed to the training phase once they answered both questions correctly.

In the training phase, participants observed a pseudorandomized sequence of 32 trials separated into 2 blocks. Each block of 16 trials was fully randomised within each block and consisted of 9 *a* trials, 3 *b* trials, 3 *c* trials, and 1 *d* trial. This trial structure contains a high outcome (75%) and cue (75%) density which is typically used to generate a causal illusion (Barberia et al., 2019; Blanco et al., 2013). Within each trial, participants were presented with either an on or off light for 1.25 s, followed by either an image of electric shock accompanied with the text “Mr X was shocked!” or a blank space with the text “Mr X was not shocked,” displayed below the light cue. The cue and outcome were presented on the screen together for 1.25 s before a prompt appeared, instructing participants to press the spacebar to continue to the next trial.

**Figure 1.**
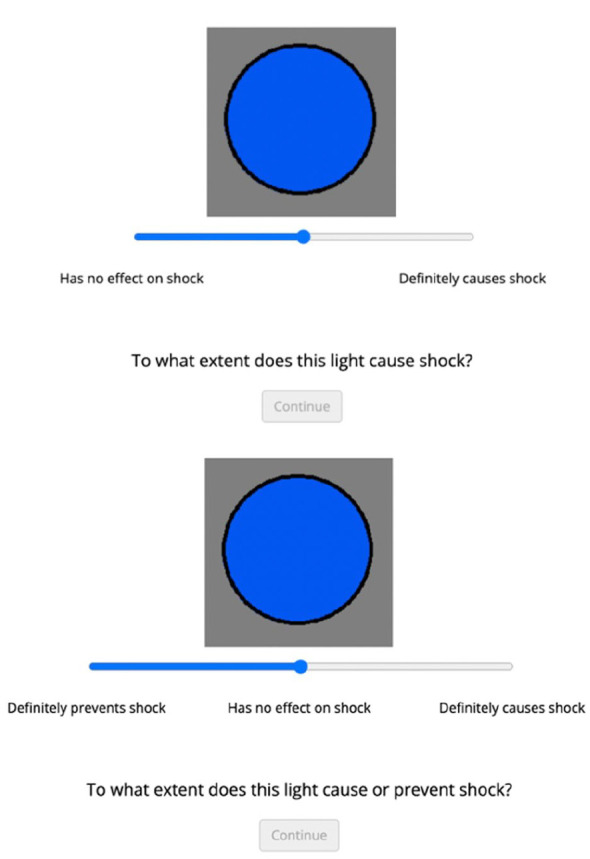
Causal rating questions in Experiment 1. *Note*: Causal rating questions for the unidirectional condition are in the top panel and questions for the bidirectional condition are in the bottom panel.

At the end of training, participants were asked to make a causal judgement rating, in which the on light was displayed along with a sliding response scale. The unidirectional group was asked “To what extent does this light cause shock?,” and the response scale was labelled “Has no effect on shock” at the left extreme and “Definitely causes shock” at the right extreme (see Figure 1, top panel). The bidirectional group was asked “To what extent does this light cause or prevent shock?,” and the response scale was labelled “Definitely prevents shock” at the left extreme, “Has no effect on shock” in the centre, and “Definitely causes shock” at the right extreme (see Figure 1, bottom panel). Participants made a response by dragging the slider to a point relative to the labels on the scale to indicate their causal beliefs. For data analysis, responses were converted to numerical ratings ranging from −100 to 100 for the bidirectional scale and 0 to 100 for the unidirectional scale. Scaling participants’ ratings in this way allowed us to observe and compare responses in both groups despite the additional response range on the bidirectional scale. Following the causal judgement question, both groups responded to two frequency questions that asked them to predict how many shocks Mr X would experience if the light was *on* for 100 observations as well as how many shocks Mr X would experience if the light was *off* for 100 observations. These frequency questions allowed us to measure participants’ perceived conditional probabilities of shock when the cue was present or absent (Barberia et al., 2019).

### Results and discussion

**Figure 2.**
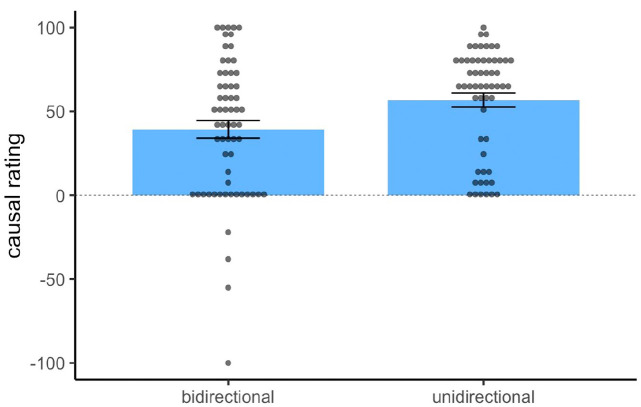
Mean and individual causal ratings for the target cue in Experiment 1. *Note.* Blue bars indicate the group means, and error bars denote standard error of the mean. The dashed line represents the normative response of 0.

All data cleaning was conducted using R (R Core Team, 2021). Subsequent analyses were conducted using R using the ez package (Lawrence, 2016) as well as jamovi (The jamovi project, 2022). Of the 119 participants recruited, 62 participants were randomly allocated to the bidirectional group and 57 to the unidirectional group. As shown in Figure 2, both groups provided positive causal ratings overall, and a one-sample *t*-test using the normative value 0 (label point for “Has no effect on shock”) as the test value reached significance for both the bidirectional group, t(61) = 7.41, *p* < .001, *d* = .94, and the unidirectional group, *t*(56) = 13.60, *p* < .001, *d* = 1.80, thus demonstrating that both groups showed an illusory causation effect regardless of the rating scale provided. Critically, however, the unidirectional group gave substantially higher causal ratings (*M* = 56.79, *SD* = 31.56) than the bidirectional group (*M* = 39.29, *SD* = 41.73). A Welch’s *t*-test was used to compare causal ratings between groups, as the assumption of equal variance in a conventional Student’s t-test would unlikely hold given the manipulation of rating scales in this experiment. The t-test yielded a significant effect, t(112.9) = 2.56, *p* = .011, *d* = .47, confirming that a stronger causal illusion was observed in the unidirectional group compared to the bidirectional group.

**Figure 3.**
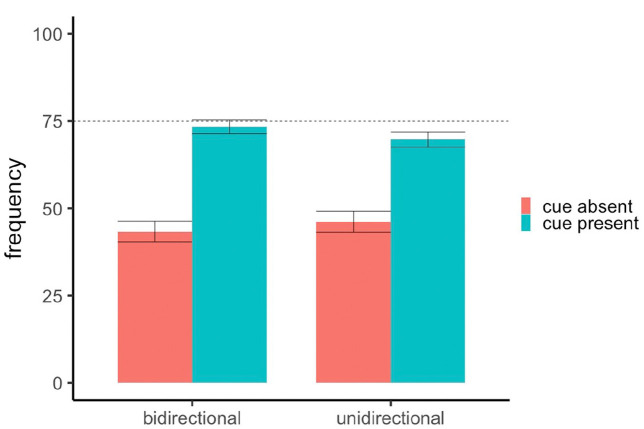
Mean frequency ratings in Experiment 1. *Note.* Error bars denote standard error. The dashed line represents the normative response of 75.

A one-sample t-test comparing all participants’ average responses to both frequency questions (*M* = 58.2, *SD* = 13.9) to the normative response of 75 (i.e., 75% outcome density) found that overall, participants significantly underestimated outcome density, *t*(118) = 13.18 *p* < .001, *d* = 1.21. As can be seen in Figure 3 this underestimation is primarily driven by participants’ frequency predictions for cue-absent trials whereas participants were fairly accurate at predicting the conditional probability of the shock when the cue was present. An ANOVA with rating scale (unidirectional vs. bidirectional) as the between-subjects factor and frequency question type (cue-present vs. cue-absent) as the within-subjects factor revealed a main effect of question type, *F*(1,117) = 111.08, *p* < .001, *d* = 1.36, where predictions were higher for cue-present trials (*M* = 71.5, *SD* = 15.7) than cue-absent trials (*M* = 44.8, *SD* = 23.0). From Figure 3, there is no observable difference in frequency ratings for cue-present and cue-absent trials between the bidirectional (*M* = 73.4, *SD* = 15.2; *M* = 43.4, *SD* = 23.3) and unidirectional groups (*M* = 69.7, *SD* = 16.2; *M* = 46.2, *SD* = 22.6). Indeed, the between-group comparison did not reach significance, *F*(1,117) < 1, nor did the interaction between -group and question type, *F*(1,117) = 1.62, *p* = .206, *d* = .33. This analysis implies that unlike their causal judgements, the presentation of a unidirectional or bidirectional scale did not influence participants’ subsequent reporting of the outcome distribution they had observed.

Participants’ frequency ratings can also be used to infer their perceptions of the trial frequencies and, therefore, compute each individual’s implied Δ*P* (see Table 1 and Equation 1). As expected from their frequency ratings and causal ratings, a positive Δ*P* was calculated for both the bidirectional (*M* = 0.300, *SD* = 0.297) and unidirectional groups (*M* = 0.235, *SD* = 0.253). Plots of mean implied Δ*P*s can be found in the Supplementary Materials. A Student’s *t*-test found that mean Δ*P*s did not significantly differ between groups, *t*(117) = 1.27, *p* = .206, *d* = 0.23. This lack of difference further demonstrates that presenting different causal rating scales did not affect participants’ reporting of their observed outcome probabilities.

**Figure 4.**
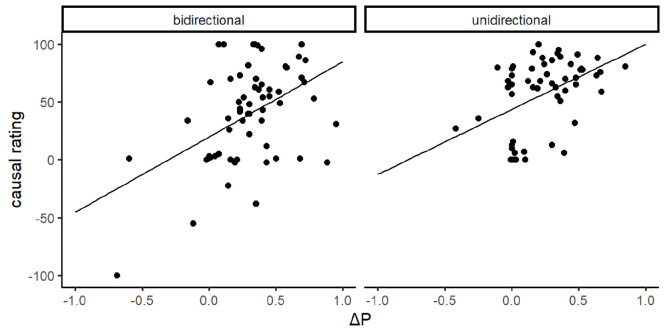
Scatterplot of implied Δ*P* compared to participants’ causal ratings in Experiment 1 with regression lines.

Figure 4 displays the relationship between participants’ causal ratings and their implied Δ*P*s. As suggested by the figure, there was a significant positive correlation in both the bidirectional group, *r* = .46, *p* < .001, and the unidirectional group, *r* = .45, *p* < .001. These findings imply that despite both groups underestimating the conditional probability of the outcome in the absence of the cue, and despite the impact of the rating scale manipulation, participants’ causal ratings still covaried with their implied Δ*P*.

### Discussion

In Experiment 1, we successfully replicated the illusory causation phenomenon in a typical causal learning paradigm with a high cue and outcome density. The strength of the illusion in the unidirectional group was comparable to other illusory causation studies that employed similar cue and outcome densities and a unidirectional rating scale (*M* = 50.6, Barberia et al., 2019; *M* = 55.3, Blanco & Matute, 2020). The bidirectional group, however, reported a significantly smaller causal illusion. This difference in causal ratings existed despite no group differences in frequency ratings, confirming that both groups processed frequency information from training trials in the same way. We may therefore attribute the difference in the strength of causal illusions between groups to the different rating scales provided during test. In particular, the bidirectional scale appears to provide a more conservative measure of participants’ causal judgements.

Another interesting finding from Experiment 1 is that both groups underestimated outcome frequency in the absence of the cue. This result is consistent with findings reported by Barberia et al. (2019), who also found that participants more accurately estimated outcome frequency in the presence of the cue and underestimated outcome frequency in the absence of the cue. Nonetheless, we did find that participants’ implied Δ*P*s correlated with their causal ratings. This suggests that their knowledge of conditional probabilities is playing a role when making causal judgements, although it is possible that frequency responses were influenced by participants’ causal ratings given the presentation order of the questions.

These findings have implications for theories of illusory causation. In particular, low ratings for the frequency of the outcome in the absence of the cue are consistent with causal judgement models which posit that people weigh *a* and *b* trials more than *c* and *d* cells when making causal judgements (White, 2003a, 2003b). By underweighting information from cue-absent trials, participants may fail to make the crucial inference that the outcome is equally likely regardless of the cue’s presence. However, due to the high cue density in our task and in Barberia et al. (2019), there were in fact more opportunities to learn the probability of the outcome on cue-present trials compared with cue-absent trials. Specifically, there were three times as many cue-present trials in our training phase compared to cue-absent trials. As such, we are unable to conclude whether the underweighting of *c* and *d* trials is due to an attentional or cognitive mechanism whereby participants discount cue-absent trials, or whether it simply reflects incomplete learning about cue-absent trials. Experiment 2 aimed to address this issue by providing participants an equal opportunity to learn the probability of the outcome on both cue-present and cue-absent trials.

## Experiment 2

In Experiment 2, we aimed to extend our investigation of the effect of rating scales on causal judgements by comparing the two scales in the same task as Experiment 1 but with 50% cue and outcome densities. These densities can be seen as neutral, in the sense that there are equal numbers of *a, b, c*, and *d* trials. Such a procedure will test whether the scale differences seen in Experiment 1 are unique to high cue and outcome density conditions. An equal cue and outcome density design will also clarify what mechanisms are producing participants’ frequency responses that we observed in Experiment 1. The design of Experiment 2 presents participants with an equal opportunity to learn the conditional probabilities of the outcome occurring on both cue-present and cue-absent trials. As such, Experiment 2 will reveal whether the causal illusions observed in Experiment 1 reflect underweighting, or less opportunity to learn about *c* and *d* trials.

### Method

The Method for Experiment 2 was the same as Experiment 1, unless otherwise specified below.

#### Participants

A total of 240 participants were recruited on the Prolific platform (104 female, 131 male, 4 non-binary, *M* age = 30.30, *SD* age = 8.97). The task completion time was expected to be 10 min as observed in Experiment 1 and participants were paid £1.50 for participating (a rate of £9.00/hr). A larger sample size was used for Experiment 2 in anticipation of a smaller effect size as a result of the equal-density design. Participants were randomly allocated into the unidirectional or the bidirectional group.

#### Procedure

Experiment 2 followed the same procedure as Experiment 1, except for the change in cell frequencies for the 2 training blocks. Participants now observed a 50% cue and 50% outcome density trial structure. That is, participants saw two training blocks, each consisting of 4 *a* trials, 4 *b* trials, 4 *c* trials, and 4 *d* trials in randomised order, before making causal judgements and frequency ratings as in Experiment 1.

### Results and discussion

**Figure 5.**
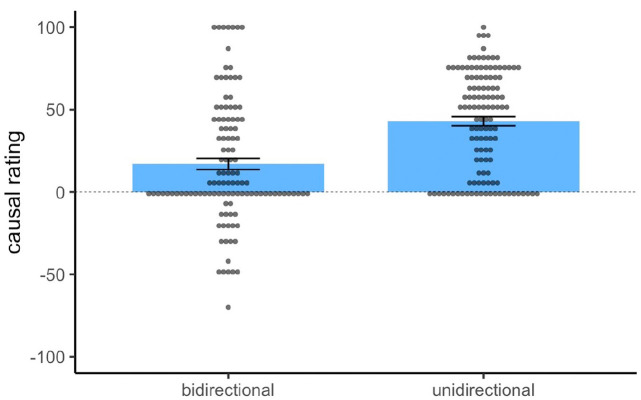
Mean and individual causal ratings for the target cue in Experiment 2. *Note.* Blue bars indicate the group means, and error bars denote the standard error of the mean. The dashed line represents the normative response of 0.

Of the 240 participants recruited, 118 were randomly allocated to the bidirectional group, and 121 were randomly allocated to the unidirectional group. One participant’s data file was lost due to technical issues during the experiment and was excluded from analysis. As shown in Figure 5, both groups again provided positive causal ratings overall. However, both group means were lower compared to Experiment 1, as expected given the lower cue and outcome densities of Experiment 2. Nonetheless, a one-sample t-test comparing causal ratings to 0 reached significance for both the bidirectional group, *t*(117) = 5.00, *p* < .001, *d* = 0.46, and the unidirectional group, *t*(120) = 15.44, *p* < .001, *d* = 1.40. These results demonstrate that a substantial illusory causation effect was still observed for both groups in Experiment 2, despite the lower means, particularly in the bidirectional group (*M* = 17.0, *SD* = 37.0). Critically, the unidirectional group gave significantly higher causal ratings (*M* = 43.0, *SD* = 30.6) than the bidirectional group, Welch *t*(226.7) = 5.89, *p* < .001 *d* = .76, which shows that the causal illusion was again larger in magnitude when rated on a unidirectional scale. Figure 5 also shows a substantial number of negative ratings made by participants in the bidirectional group.

**Figure 6.**
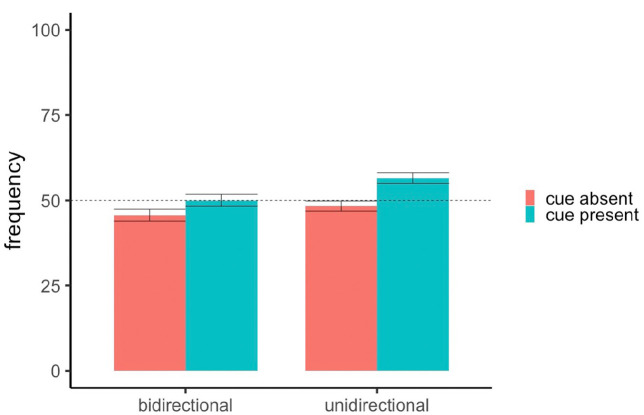
Mean frequency ratings for cue-present and cue-absent trials in Experiment 2. *Note.* Error bars denote standard error. The dashed line represents the normative response of 50.

Figure 6 shows participants’ frequency predictions in Experiment 2. Critically, the underestimation of outcome frequency for cue-absent trials observed in Experiment 1 was not replicated in Experiment 2. In fact, both groups made ratings that were relatively close to the normative response of 50 (i.e., 50% outcome density) for both cue-present and cue-absent trials. A one-sample t-test comparing all participants’ average frequency ratings (*M* = 50.2, *SD* = 12.2) to 50 did not find a significant effect, *t*(238) = 0.20, *p* = .841, *d* = .01. An ANOVA found a significant main effect of group, *F*(1,237) = 8.80, *p* = .003, *d* = .256 where the unidirectional group (*M* = 52.4, *SD* = 17.2) gave higher ratings than the bidirectional group (*M* = 47.8, *SD* = 19.3). There was also a significant main effect of question type, *F*(1,237) = 13.16, *p* < .001, *d* = .349 where frequency ratings to cue-present trials (*M* = 53.3, *SD* = 18.1) were higher than cue-absent trials (*M* = 47.0, *SD* = 17.9). The significant differences in frequency ratings seem to be driven primarily by the slight overestimation of the outcome on cue-present trials in the unidirectional group, although the interaction between group and question type was not significant *F*(1,237) = 1.24, *p* = .267, *d* = .214. As such, although participants made more accurate frequency ratings overall when presented with a 50% cue and outcome density task, we still observed slightly higher ratings for cue-present trials compared to cue-absent trials, and slightly higher overall ratings in the unidirectional group.

**Figure 7.**
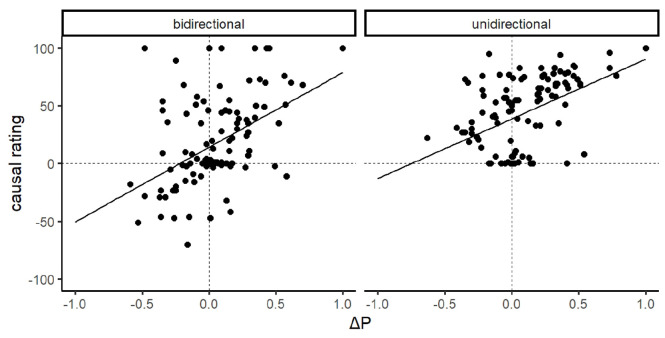
Scatterplot of implied Δ*P* compared to participants’ causal ratings in Experiment 2 with regression lines.

Likewise, participants’ implied Δ*P*s were also closer to the normative values in the bidirectional (*M* = 0.044, *SD* = 0.267) and unidirectional (M = 0.082, *SD* = 0.268) groups, as expected from their frequency ratings. Again, there was no group difference in Δ*P*s, *t*(237) = 1.11, *p* = .267, *d* = .14, further demonstrating that although both groups differed in their causal ratings, the type of rating scale presented did not affect reporting of their perceived trial frequencies. Figure 7 displays the relationship between participants’ causal ratings and their implied Δ*P*s in Experiment 2. Again, there was a significant positive correlation in both the bidirectional group, *r* = .47, *p* < .001, and the unidirectional group, *r* = .45, *p* < .001. As such, Experiment 2 replicates the findings in Experiment 1 implying that participants use Δ*P* to some degree as a rule to make causal judgements.

## General discussion

Over two experiments, we compared the effect of unidirectional and bidirectional rating scales in assessing participants’ causal judgements in a zero contingency learning task. We observed causal illusions with both scales, regardless of whether cue and outcome density were high (both 75%, Experiment 1) or neutral (both 50%, Experiment 2). However, mean causal ratings were significantly higher (less normative) in the group that gave ratings on a unidirectional scale compared to the group that gave ratings on a bidirectional scale.

There are at least three factors that may account for these group differences in causal ratings. First, the nature of the unidirectional rating scale censored negative responses, restricting variance in participants’ causal judgements to be distributed among the positive section of the scale range (see Figures 4 and 7). This pattern was most apparent in Experiment 2, where a substantial number of negative ratings were observed in the bidirectional group, but not of course in the unidirectional group, even though the groups had been exposed to identical training and presumably had similar causal beliefs. Second, the normative response of zero was located in the middle of the scale for the bidirectional group compared to an extreme the scale in the unidirectional group. Thus participants in the unidirectional group may have been less willing to report a zero contingency compared to the bidirectional group because it was located at a scale extreme (Baumgartner & Steenkamp, 2001). In fact, for a normative mean causal rating to be observed in the unidirectional group, *all* participants would have to give a score of zero, which lies at one extreme of their rating scale and is likely to be avoided by most participants. Finally, participants who were confused or simply responding randomly would tend to produce a mean causal rating of 50 on a unidirectional scale (a strongly non-normative response), but a mean of 0 on a bidirectional scale. All of these factors suggest that a unidirectional scale produces higher causal ratings through mechanisms that are not related to participants’ actual causal beliefs; in other words, a unidirectional scale exaggerates the true size of causal illusions. Nonetheless, a robust causal illusion was observed in participants who used a bidirectional rating scale in both experiments. This scale is not affected by any of the artefacts discussed above, confirming that the illusory causation effect is a genuine phenomenon.

An additional goal in Experiment 2 was to determine whether this illusion could be attributed to an underweighting of *c* and *d* trials (White, 2003a, 2003b) or to a lack of opportunity to learn the probability of the outcome on these trials when cue density is high. By reducing cue density to 50% in Experiment 2, we gave participants an equal opportunity to learn the conditional probability of the outcome on both cue-present and cue-absent trials. This change resulted in frequency ratings and Δ*P*s that were indeed closer to normative values (i.e., 50%; 0) compared to the underestimation of outcome frequency on cue-absent trials observed in Experiment 1. Despite participants giving more normative frequency ratings, however, an illusion was still observed in Experiment 2 for both groups. As such, causal illusions do not appear to stem entirely from an inability or lack of opportunity to learn the conditional probability of the outcome occurring on cue-absent trials (cells c and d). Rather, the illusion may be driven by the inability to accurately integrate learned conditional probabilities to infer causal relationships. The positive correlation observed in both experiments between causal ratings and implied Δ*P* suggests that participants do take into account cue-absent trials to some extent. However, a substantial number of participants gave ratings well above zero on both rating scales, suggesting that they are not integrating conditional probability information in a way that completely matches Δ*P*. This interpretation is supported by previous studies showing that educational interventions explaining the logic of randomised controlled clinical trials (e.g., comparing outcome probabilities between when the cause is present and absent) can successfully reduce causal illusions (Barberia et al., 2013; Goldwater et al., 2020).

The present results have several implications for our interpretation of the existing illusory causation literature and how we should investigate the phenomenon moving forward. Given that illusory causation paradigms are presented as an analogue of how people come to form fallacious beliefs, it is critical that we are accurately capturing participants’ causal judgements within our experiments before applying findings to real-world contexts. The majority of published experiments employing illusory causation paradigms have used unidirectional rating scales (e.g., Barberia et al., 2019; Blanco et al., 2011; Matute et al., 2011), which may have complicated identification of the conditions that generate causal illusions. Our results also have implications for studies that aim to eliminate or reduce causal illusions. For example, Barberia et al. (2019) found that extended training reduced the strength of causal illusions but did not completely eliminate them. It is possible that the efficacy of such manipulations could be understated when measured with unidirectional rating scales, similar to our findings in Experiment 2 where a reduction in cue and outcome density appeared to produce a greater decrease in causal ratings for the bidirectional group compared to the unidirectional group.

In conclusion, our results reveal that although illusory causation is a genuine phenomenon, a proportion of the effect may be due to the use of a unidirectional rating scale. Participants’ true belief in a causal relationship between illusory cues and outcomes in these paradigms may therefore be weaker than previous experiments imply. Our results suggest that the bias associated with unidirectional rating scales is a general one that is independent of cue and outcome density effects. As such, we recommend using bidirectional scales in future causal illusion or contingency learning research to capture a more accurate and unbiased representation of participants’ causal judgements.

## Supplemental Material

sj-docx-1-qjp-10.1177_17470218231175003 – Supplemental material for Unidirectional rating scales overestimate the illusory causation phenomenon Supplemental material, sj-docx-1-qjp-10.1177_17470218231175003 for Unidirectional rating scales overestimate the illusory causation phenomenon by David W Ng, Jessica C Lee and Peter F Lovibond in Quarterly Journal of Experimental Psychology
