# Supplementary material for: Unidirectional rating scales overestimate the illusory causation phenomenon
Source: Q J Exp Psychol (Hove). 2023 May 23;77(3):551–62. doi: 10.1177/17470218231175003 (PMC10880420; doi:10.1177/17470218231175003)
Supplement: sj-docx-1-qjp-10.1177_17470218231175003 – Supplemental material for Unidirectional rating scales overestimate the illusory causation phenomenon [file sj-docx-1-qjp-10.1177_17470218231175003.docx]

Supplementary Material for:

**Unidirectional rating scales overestimate the illusory causation phenomenon**

David W. Ng, Jessica C. Lee, Peter F. Lovibond

University of New South Wales

***Corresponding Author:***

Ng, David Wen Hao

University of New South Wales

Sydney, New South Wales 2052

Australia

<https://orcid.org/0000-0002-1787-0841>

david.ng2@unsw.edu.au

**Task Instructions for Experiment 1 and 2**


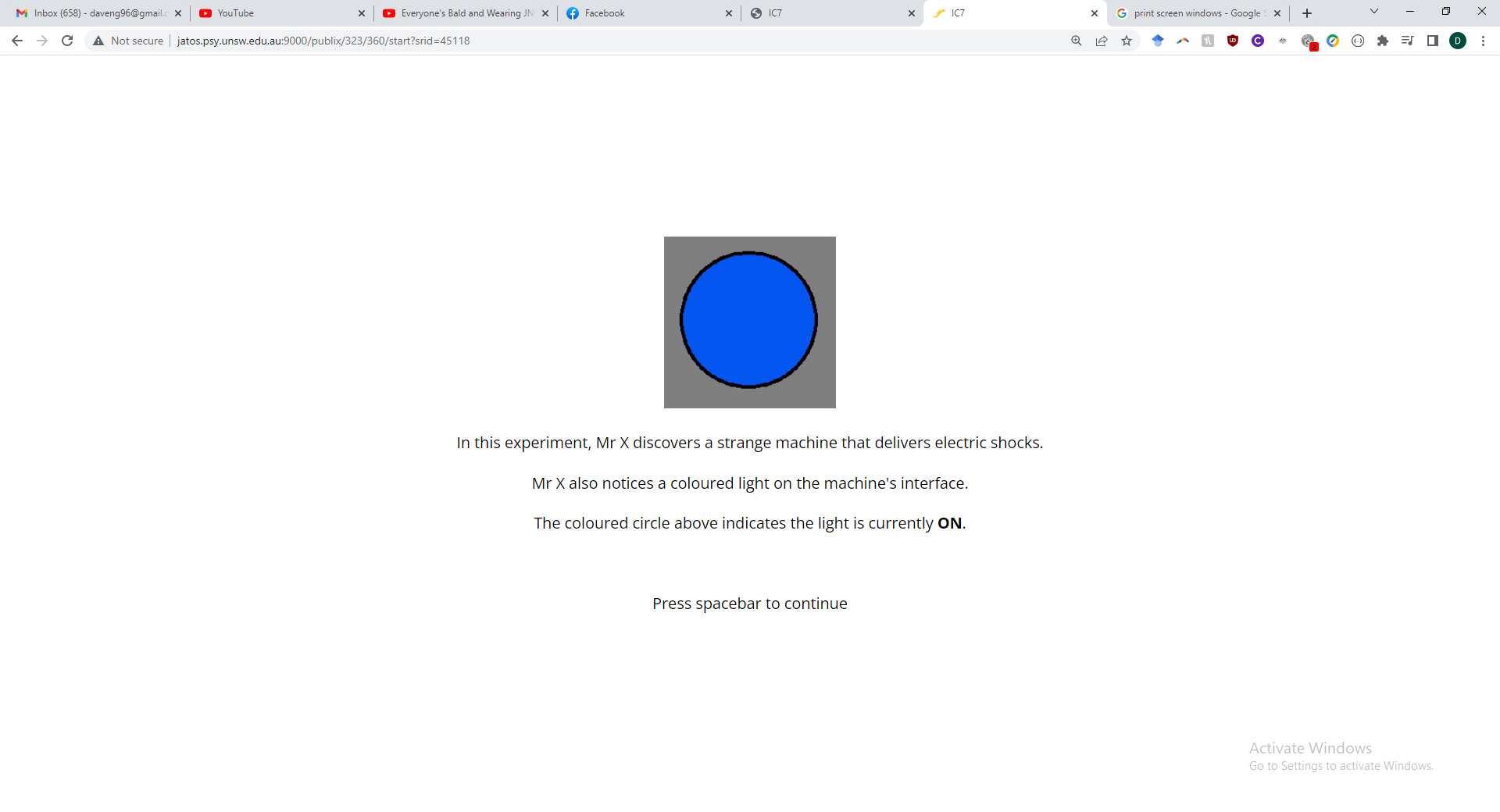


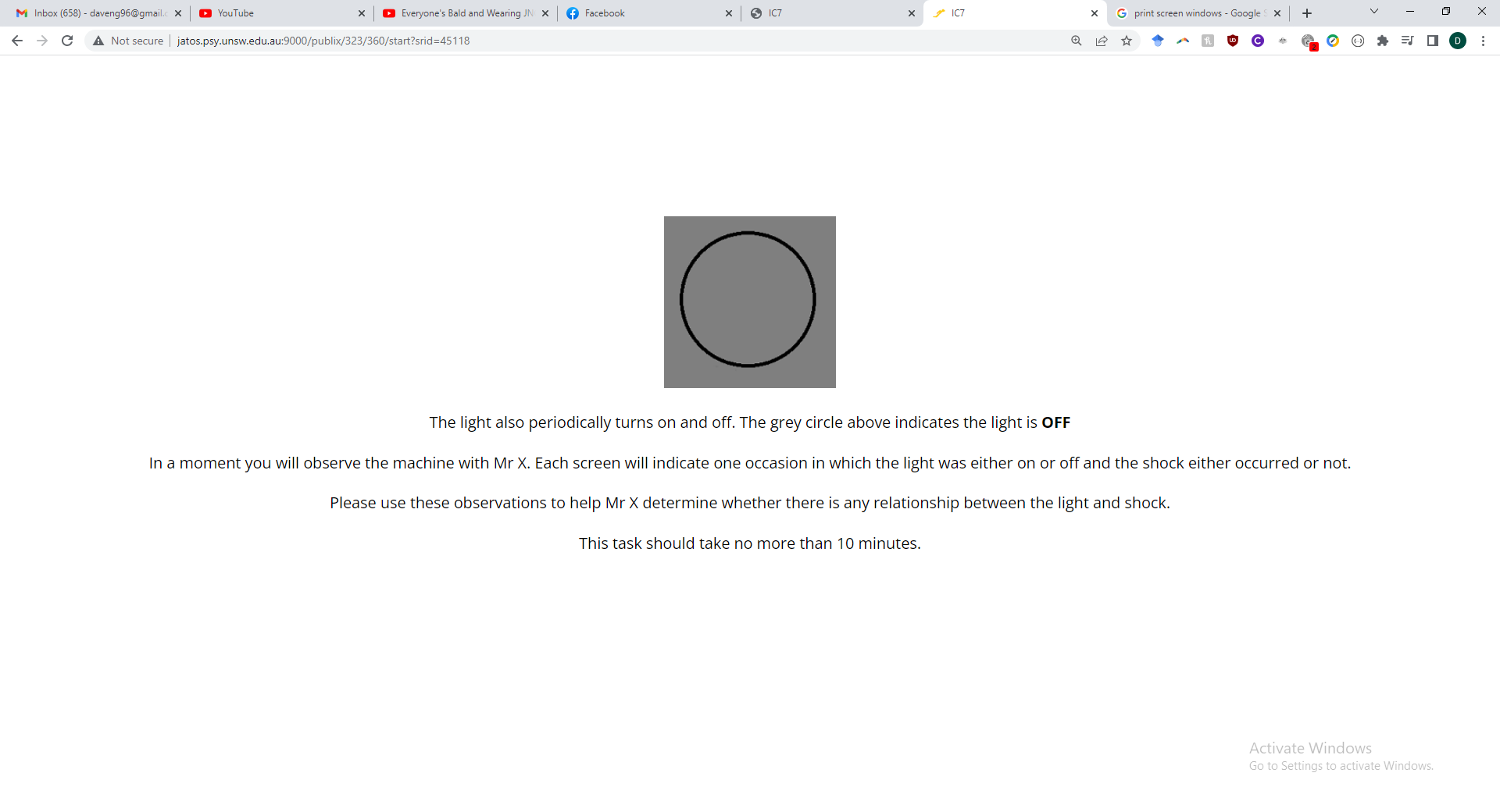


**Mean Implied ΔPs in Experiment 1**

**
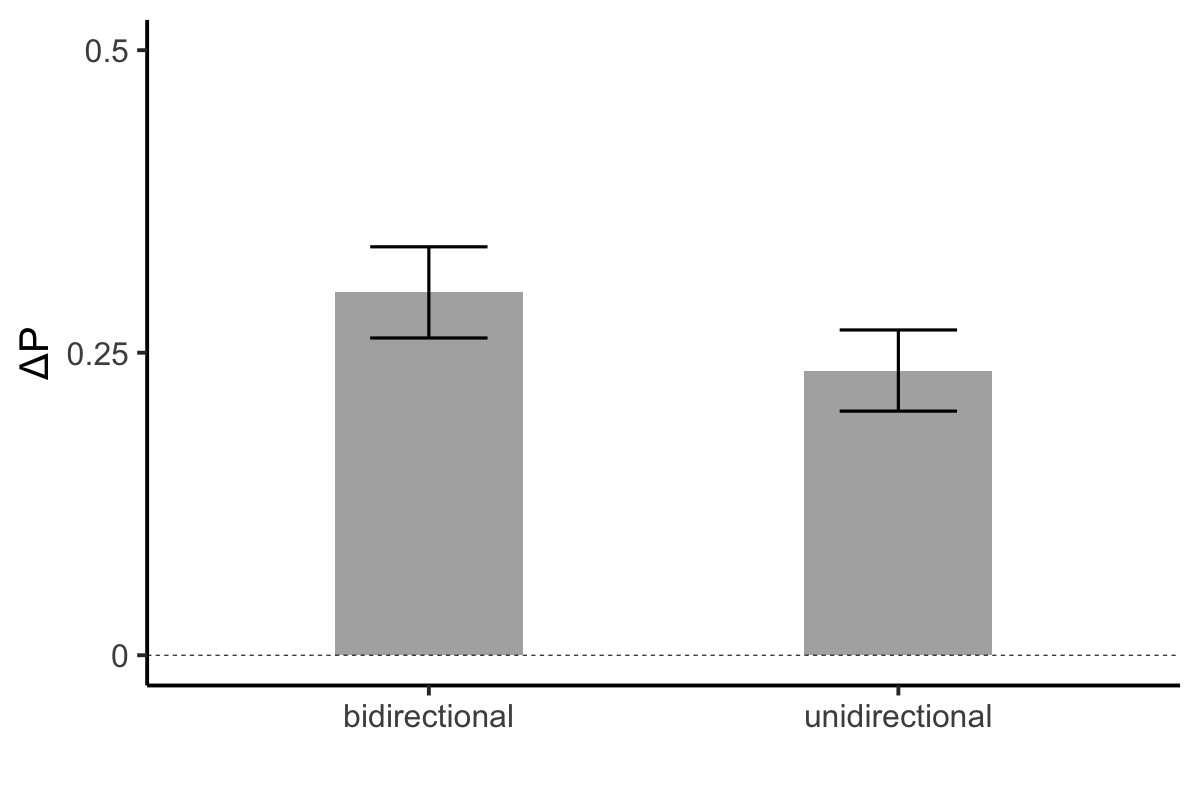
**

**Mean Implied ΔPs in Experiment 2**

**
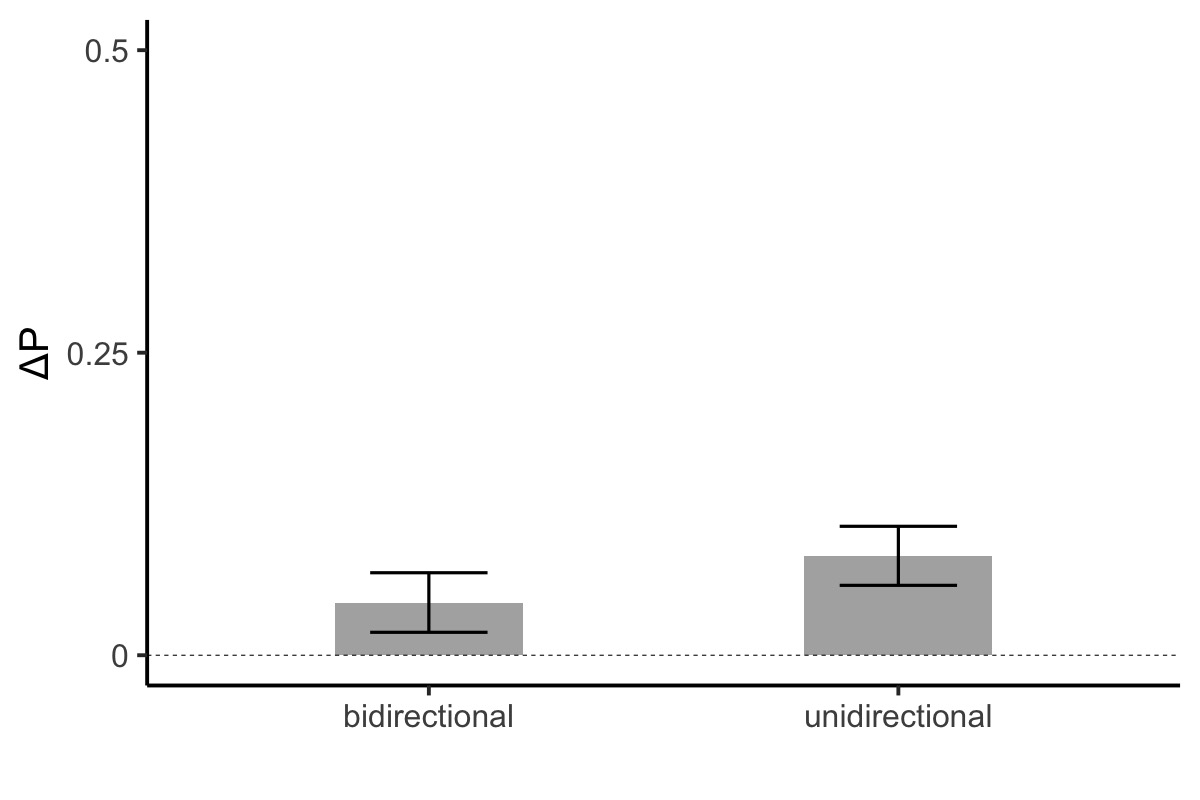
**
